# Assessing the Geographic Context of Risk Perception and Behavioral Response to Potential Ebola Exposure

**DOI:** 10.3390/ijerph16050831

**Published:** 2019-03-07

**Authors:** Eric Shook, Andrew Curtis, Jacqueline Curtis, Gregory Gibson, Anthony Vander Horst, Virginia Little, Christopher Woolverton

**Affiliations:** 1 Department of Geography, Environment, and Society, University of Minnesota, 414 Social Sciences Building, 267 19th Ave S, Minneapolis, MN 55455, USA; 2 Department of Geography, GIS, Health & Hazards Lab, Kent State University, Kent, OH 44240, USA; acurti13@kent.edu (A.C.); jcurti21@kent.edu (J.C.); 3 Department of Sociology, Kent State University, Kent State University, Kent, OH 44242, USA; ggibson4@kent.edu (G.G.); avanderh@kent.edu (A.V.H.); vlittle@kent.edu (V.L.); 4 College of Public Health, Kent State University, Kent, OH 44242, USA; cwoolver@kent.edu

**Keywords:** ebola virus disease, risk reduction behavior

## Abstract

The 2014–2016 Ebola Virus Disease (EVD) epidemic outbreak reached over 28,000 cases and totaled over 11,000 deaths with 4 confirmed cases in the United States, which sparked widespread public concern about nationwide spread of EVD. Concern was elevated in locations connected to the infected people, which included Kent State University in Kent, Ohio. This threat of exposure enabled a unique opportunity to assess self-reported knowledge about EVD, risk perception, and behavior response to EVD. Unlike existing studies, which often survey one point in time across geographically coarse scales, this work offers insights into the geographic context of risk perception and behavior at finer-grained spatial and temporal scales. We report results from 3138 respondents comprised of faculty, staff, and students at two time periods. Results reveal increased EVD knowledge, decreased risk perception, and reduction in protective actions during this time. Faculty had the lowest perceived risk, followed by staff and then students, suggesting the role of education in this outcome. However, the most impactful result is the proof-of-concept for this study design to be deployed in the midst of a disease outbreak. Such geographically targeted and temporally dynamic surveys distributed during an outbreak can show where and when risk perception and behaviors change, which can provide policy-makers with rapid results that can shape intervention practices.

## 1. Introduction

During the fall of 2014, four Ebola Virus Disease (EVD) events in the United States caused both national and localized concern [1,2,3]. To the authors’ knowledge, few attempts were made to surveil local “at risk” populations from any of these events. Yet such contemporaneous insight could help shed light on the link between perceptions and protective actions within a major epidemic [4,5].

Perception of risk, such as potential exposure to EVD, may motivate individuals to modify their behavior and take protective actions to try to reduce risk of infection [4,5,6]. As a whole, these individuals help to protect the susceptible population through improved personal hygiene, social distancing, getting vaccinated, wearing condoms, or modifying burial practices to eliminate physical contact with the deceased, thereby altering the progression of a disease outbreak [5,6,7,8,9]. However, the adoption of preventative actions can be difficult to capture in the midst of an epidemic. 

This study surveyed individuals associated with Kent State University (KSU), including students, staff, and faculty. KSU is a public university in Kent, Ohio, which had indirect contact with a soon-to-be symptomatic healthcare worker who had been treating an EVD-infected patient in Dallas, Texas. The healthcare worker traveled to Northeast Ohio near KSU while in a pre-symptomatic stage. She returned to Dallas, Texas, to become the third person in the US to be diagnosed with EVD. We conducted an online survey to gauge initial and current knowledge about the disease and EVD-related risk perception and behavior response across the Kent State University system. KSU is the largest employer in the county, with tens of thousands of students, staff, and faculty, and as such the KSU community represents a major segment of the population in the surrounding area that could have had direct or indirect contact with the healthcare worker. It was also a population that could rapidly be surveyed, because the research team had the ability to email every student, staff, and faculty member in the eight-campus system, representing a unique opportunity during a critical time of potential EVD exposure. The survey instrument was disseminated rapidly to capture change in risk perception and behavior response during a major epidemic, which provides insight to human behavior during this crucial period that is often intermixed with fear and misperception [10].

## 2. Materials and Methods 

All students, staff, and faculty in the KSU system were emailed an online survey yielding 4313 responses from 8 campuses across Northeast Ohio (IRB-protocol #14-526). The response rate was 7.46%. There were no incentives for participating in the survey. On October 29, 2014, the survey was sent by email to all employees, faculty, and students. Following this email, potential respondents were sent two reminders on November 3 and November 13. The survey instrument included questions related to role at the university (student, staff, faculty, and other), sex (male, female, other), and a question asking participants to identify their primary campus. We report results from 3138 respondents from KSU main campus, which was closest to where the healthcare worker stayed in Northeast Ohio and had the strongest connection to the (by then) symptomatic health care worker. Of these survey respondents, 77.5% were students, 11.9% staff, 7.3% faculty, and 3.2% reported other (29.3% were male, 69.3% female, 0.3% reported other).

The first survey was sent 2 weeks after KSU issued an alert about EVD on October 15, 2014 [11], followed by two reminders. Respondents were asked to rate their level of knowledge about Ebola, perceived risk of contracting Ebola, and behavioral responses for two time periods: on October 15, when they first learned about the connection between Ebola and KSU (hereafter referred to as “initially”), and at the time of taking the survey, at least 2 weeks later (hereafter referred to as “currently”).

Initial and current knowledge were assessed using two questions with respondents “rating their level of Ebola knowledge (e.g., what it is, how it is transmitted, how to protect yourself)” from (1) no knowledge at all, to (5) high level of knowledge. Initial and current perceived risk was assessed using two questions. Respondents were asked to "rate your risk of contracting Ebola" from (1) very low, to (5) very high.

Respondents were asked whether or not they had taken protective actions resulting from concerns for contracting EVD for both initial and current periods. Protective actions included: (1) washing hands more frequently; (2) using hand sanitizer more frequently; (3) avoiding public transportation; (4) avoiding school or work; (5) avoiding people who are coughing or sneezing; and (6) avoiding large gatherings of people. Two indicator scores for behavior response to EVD were calculated by totaling the number of personal hygiene actions and public avoidance actions similar to calculated scores in other studies [12]. Public avoidance measures were surveyed to gauge reported cases of individuals avoiding large social gatherings owing to reduce their perceived risk of potential infection.

## 3. Results

Reported high rates of knowledge (rated 45) increased from 50% initially to 78% (Table 1). Individuals having no knowledge reduced from 4% to 0.6%. Reported mean knowledge rating increased from 3.51 (SD = 1.1) initially to 4.14 (SD = 0.87), with 1.36% answering “do not know or prefer not to answer (unrated)”. A paired-sample t-test was conducted to compare respondents who rated their initial and current knowledge. There was a significant difference in level of initial and current self-reported knowledge; t (3082) = −36.84, *p* < 0.001.

Perceived risk of contracting Ebola fell from 22% rating moderate to very high risk (3–5) to 5%. A paired-sample *t*-test was used to find a significant difference in self-reported rating of initial and current perceived risk; t (3027) = 28.28, *p* < 0.001, in which perceived risk fell from a mean of 1.77 (SD = 1.11) initially to 1.24 (SD = 0.62), with 2.6% unrated responses. Women were more likely to perceive higher risk (3–5) initially (23%) compared to men (19%). There was a statistically significant difference between students, staff, faculty, and others for initial perceived risk as determined by one-way ANOVA; F (3, 3079) = 8.36, *p* < 0.001. Students (23%) and staff (22%) perceived higher risk (3–5) initially compared to faculty (10%).

The majority of respondents reported increased hand washing (57%) initially. Nearly 47% of respondents reported using hand sanitizer more frequently initially. A mean score of 1.04 (SD = 0.91) was obtained for personal hygiene (range 0–2). Public avoidance ranged from 36% avoiding those coughing or sneezing to 3.9% avoiding school or work (Table 2). Respondents also avoided large gatherings of people (19%) and public transportation (18%). A mean score of 0.77 (SD = 1.10) was obtained for public avoidance (range 0–4). We observe a decrease in the adoption for all protection actions from the initial period to the current period, but note that almost half of respondents (up to 48.6%) were still taking protective actions when the survey was taken several weeks after learning about potential EVD exposure, including a smaller percentage who continued to avoid gatherings, public transportation, and school or work.

Initial perceived risk was positively associated with adoption of protective actions, both initially and currently. Self-reported knowledge had very weak, negative associations with perceived risk and adoption of protective actions. Adoption of personal hygiene and public avoidance actions were positively associated. Interestingly, initial perceived risk was more strongly associated with initial and current adoption of protective actions when compared to current perceived risk or EVD knowledge (Table 3).

## 4. Discussion

A largely under-researched aspect of epidemiology is how the public perceives threat and modifies behavior during a massive disease outbreak. Furthermore, much of the existing knowledge is geographically coarse in scale, such as national or statewide surveys and sampled responses for only one time period [13,14,15,16]. We report knowledge about EVD, perceived risk, and protective actions during two periods for a campus population under an EVD “watch” at a fine spatial resolution and covering two time periods. This study design should be viewed as a successful proof of concept that will enable future investigation into the geographic context of knowledge, risk perception, and behaviors in the midst of an epidemic. Insights from such work can provide public health practitioners and policy-makers with rapid and near real-time situation awareness that can shape their communication and intervention practices.

Unlike many studies conducted months after an outbreak, this survey was conducted while 164 Ohio contacts were being monitored, including 3 individuals at KSU [1]. However, we observe lower levels of concern compared to national surveys of USA residents [2,13], which may be due to an information campaign on campus, significant local and national media coverage, and educational attainment of respondents. Results suggest that initial perceived risk is a potential indicator for adopting protective actions. Overall, the study finds that this geographically concentrated population was knowledgeable about EVD, perceived little to moderate risk, and yet many adopted protective actions. More respondents adopted personal hygiene actions compared to public avoidance actions. However, it is the proof of concept that is the main contribution of this study. More geographically explicit and temporally dynamic surveys distributed and analyzed throughout a disease outbreak can show where and when knowledge, risk perception, and behaviors change.

### Limitations

This study was designed and implemented in the midst of an EVD watch for the study population, which also covered the home and work locations of the investigators. Such a context would naturally be expected to introduce limitations into any study, some of which are evident, and perhaps others that require more reflection. First, the survey collected limited demographic information to reduce the number of questions with the explicit aim of increasing the response rate. The surveyed population was restricted to a public education institution, which limits the generalizability of this study to a general population due to the higher than average education levels of the majority of respondents. The survey instrument used self-reporting of risk perception and EVD knowledge rather than a more comprehensive suite of questions used in other assessments of disease [12,17,18]. Lastly, the survey instrument used retrospective reporting (e.g., “Initially” questions), which is known to have issues with memory biases [19]. This limitation could influence the self-reported actions and perceptions in individuals. The context of this study and the limitations that resulted highlight the need for more research on how being in a hazard zone provides both unique opportunities for knowledge creation, while simultaneously introducing unique constraints to what can be accomplished under such stress.

## 5. Conclusions

Potential exposure to any life-threatening disease is a terrifying prospect for the public and for the health professionals who must contain these outbreaks. Such events generate frightening headlines and persistent media coverage [16,20], but what actually happens in situ? For those people who share geographic proximity with an infected person, what do they think and how do they behave? Answers to these questions are essential for establishing a knowledge base of what comes next. Specifically, will the risk perceptions and related behaviors adopted by those in the affected locations act to exacerbate or diminish the spread of disease? This study utilizes rare access to an entire institution during its potential exposure to EVD to offer answers to this question. It finds improved knowledge, lower perceived risk, and adoption of fewer protective actions between receiving the initial institutional alert and upon taking the survey. Women and students report higher levels of perceived risk and adopted more protective actions. Our results do not show mass panic in response to perceived Ebola exposure, but do show adoption of protective action aimed at reducing the risk of contracting EVD, including less-effective actions such as increased hand sanitizer usage. However, anecdotal evidence suggested that concern was far more elevated for those outside of the university system. Furthermore, our findings may be used to advance a number of models in public health, such as the Health Belief Model (HBM) [21], and those in other allied domains, such as the Protective Action Decision Model [22] and the Social Amplification of Risk Model [23]. Our study can advance models such as the HBM that suggests a person’s belief about susceptibility predicts adoption of preventative behavior to potentially life-threatening diseases. While decisions to adopt preventative behavior are indeed personal, people do not live in a vacuum. Our study design provides a new avenue to examine how geographic context can shape individual beliefs, perceptions, and actions, including the role that institutions play in disseminating information, lowering (or potentially heightening) perceived barriers, and instituting cues to action. By studying the fine geographic and temporal scales, this work also informs other risk perception studies, especially with regards to information barriers that occur inside and outside of an institution, and ultimately can help quantify behavior response in disease models [7,9].

## Figures and Tables

**Table 1:** Initial and current Ebola Virus Disease-related knowledge and perceived risk.

Reported Rates	Mean (SD)	High or very High (4,5)
Initial knowledge	3.51 (1.10)	50.38%
Current knowledge	4.14 (0.87)	78.33%
Initial perceived risk	1.77 (1.11)	9.43%
Current perceived risk	1.24 (0.62)	1.37%

**Table 2:** Proportion of respondents who reported taking protective actions due to concern about contracting Ebola.

Protective Action	Initially (%)	Currently (%)
More frequent hand washing	56.9%	48.6%
More frequent hand sanitizer usage	46.9%	40.3%
Avoid people who cough/sneeze	36.2%	30.2%
Avoid large gatherings of people	19.1%	11.76%
Avoid public transportation	18.1%	13.2%
Avoid school/work	3.9%	2.4%

**Table 3:** Pearson correlations between mean self-reported knowledge, perceived risk, personal hygiene score, and public avoidance score for initial and current periods.

		Initially			Currently			
		1.	2.	3.	4.	5.	6.	7.
**Initially**	1. Knowledge							
	2. Perceived risk	−0.16 ^a^						
	3. Personal hygiene score	−0.09 ^a^	0.35 ^a^					
	4. Public avoidance score	−0.09 ^a^	0.43 ^a^	0.47 ^a^				
**Currently**	5. Knowledge	0.56 ^a^	−0.10 ^a^	−0.03	−0.07 ^a^			
	6. Perceived risk	−0.08 ^a^	0.43 ^a^	0.22 ^a^	0.34 ^a^	−0.15 ^a^		
	7. Personal hygiene score	−0.07 ^a^	0.30 ^a^	0.82 ^a^	0.46 ^a^	−0.03	0.24 ^a^	
	8. Public avoidance score	−0.04 ^b^	0.34 ^a^	0.38 ^a^	0.78 ^a^	−0.07 ^a^	0.37 ^a^	0.45 ^a^

Note: ^a^ *p* < 0.001, ^b^ *p* < 0.05.
